# Controversial Role of the Immune Checkpoint OX40L Expression on Platelets in Breast Cancer Progression

**DOI:** 10.3389/fonc.2022.917834

**Published:** 2022-07-08

**Authors:** Susanne M. Rittig, Martina S. Lutz, Kim L. Clar, Yanjun Zhou, Korbinian N. Kropp, André Koch, Andreas D. Hartkopf, Martina Hinterleitner, Lars Zender, Helmut R. Salih, Stefanie Maurer, Clemens Hinterleitner

**Affiliations:** ^1^ Department of Hematology, Oncology and Cancer Immunology, Charité – Universitaetsmedizin Berlin, Corporate Member of Freie Universitaet Berlin and Humboldt-Universitaet zu Berlin, Berlin, Germany; ^2^ Berlin Institute of Health at Charité – Universitaetsmedizin Berlin, BIH Biomedical Innovation Academy, BIH Charité (Junior) (Digital) Clinician Scientist Program, Berlin, Germany; ^3^ Clinical Collaboration Unit Translational Immunology, German Cancer Consortium (DKTK), Department of Internal Medicine, University Hospital Tuebingen, Tuebingen, Germany; ^4^ Cluster of Excellence iFIT (EXC 2180) “Image-Guided and Functionally Instructed Tumor Therapies” , University of Tuebingen, Tuebingen, Germany; ^5^ Department of Hematology, Medical Oncology and Pneumology, University Medical Center of Mainz, Mainz, Germany; ^6^ Department of Obstetrics and Gynecology, University Hospital Tuebingen, Tuebingen, Germany; ^7^ Department of Gynecology and Obstetrics, University Hospital of Ulm, Ulm, Germany; ^8^ Department of Medical Oncology and Pneumology (Internal Medicine VIII), University Hospital Tuebingen, Tuebingen, Germany; ^9^ German Cancer Research Consortium (DKTK), Partner Site Tuebingen, German Cancer Research Center (DKFZ), Heidelberg, Germany; ^10^ Precision Immunology Institute, Department of Oncological Sciences, and The Tisch Cancer Institute, Icahn School of Medicine at Mount Sinai, New York, NY, United States; ^11^ Department of Cancer Biology and Genetics, Memorial Sloan Kettering Cancer Center, New York, NY, United States

**Keywords:** breast cancer, biomarker, immunotherapy, platelets, prognosis, OX40L

## Abstract

In conventional T cells, OX40 has been identified as a major costimulating receptor augmenting survival and clonal expansion of effector and memory T cell populations. In regulatory T cells, (Treg) OX40 signaling suppresses cellular activity and differentiation. However, clinical trials investigating OX40 agonists to enhance anti-tumor immunity, showed only limited success so far. Here we show that platelets from breast cancer patients express relevant levels of OX40L and platelet OX40L (pOX40L) inversely correlates with platelet-expressed immune checkpoint molecules GITRL (pGITRL) and TACI (pTACI). While high expression of pOX40L correlates with T and NK cell activation, elevated pOX40L levels identify patients with higher tumor grades, the occurrence of metastases, and shorter recurrence-free survival (RFS). Of note, *OX40* mRNA levels in breast cancer correlate with enhanced expression of anti-apoptotic, immune-suppressive, and tumor-promoting mRNA gene signatures. Our data suggest that OX40L on platelets might play counteracting roles in cancer and anti-tumor immunity. Since pOX40L reflects disease relapse better than the routinely used predictive markers CA15-3, CEA, and LDH, it could serve as a novel biomarker for refractory disease in breast cancer.

## Background

Immune checkpoints are crucial parts of inhibitory or activating immune pathways, regulating self-tolerance and immune responses. Particularly in the context of malignant disease, immune-inhibitory receptors including PD-1 or CTLA-4, are triggered to dampen anti-tumor immune responses (1–3). In contrast, co-stimulatory signaling pathways like CD27, CD40, or OX40 have been described to play a crucial role in T cell activation, proliferation, and tumor immune surveillance (4–6). An increasing number of therapeutic strategies have been exploited to block or activate the respective pathways and reinforce anti-tumor immunity (6–9). Strategies to improve co-stimulatory pathways like OX40 have shown promising anti-tumor activity in preclinical mouse models (10–12). The immune checkpoint molecule OX40 is a member of the tumor necrosis factor receptor (TNFR) superfamily. It is expressed on effector T lymphocytes following activation and promotes their differentiation, proliferation, and survival while simultaneously inhibiting the suppressive activity of regulatory T cells (13). Although monoclonal antibodies targeting OX40 are currently being investigated in early clinical trials, a successful translation of the promising preclinical data is still pending (14, 15). Of note, the expression of the cognate ligand (OX40L), also known as tumor necrosis factor superfamily member 4 (TNFSF4) on platelets has been described to be involved in cardiac inflammation and solid tumors (16, 17).

Platelets play a critical role in tumor progression, formation of metastasis, tumor immunosurveillance, and immunotherapy *via* a manifold of mechanisms, including coating of tumor cells, the release of soluble factors, e.g. TGF-ß, VEGF, and expression of immunoregulatory molecules like PD-L1, GITRL, CD40L and TACI (18–25). Here we show that the immune checkpoint molecule OX40L is frequently expressed on platelets in breast cancer and is negatively correlated with the expression of the immune checkpoint molecules GITRL and TACI on the platelet surface. In breast cancer, high levels of pOX40L were found to be associated with immune cell activation, higher tumor grades, formation of metastasis, and reduced recurrence-free survival (RFS), indicating that pOX40L might play a controversial role in tumor biology *via* orchestrating immune activation and tumorigenesis. We demonstrate that pOX40L is superior in predicting disease progression compared to routinely used markers in breast cancer.

## Results and Discussion

### Expression of OX40L on Platelets Correlates With Immune Cell Activation

**Figure 1:**
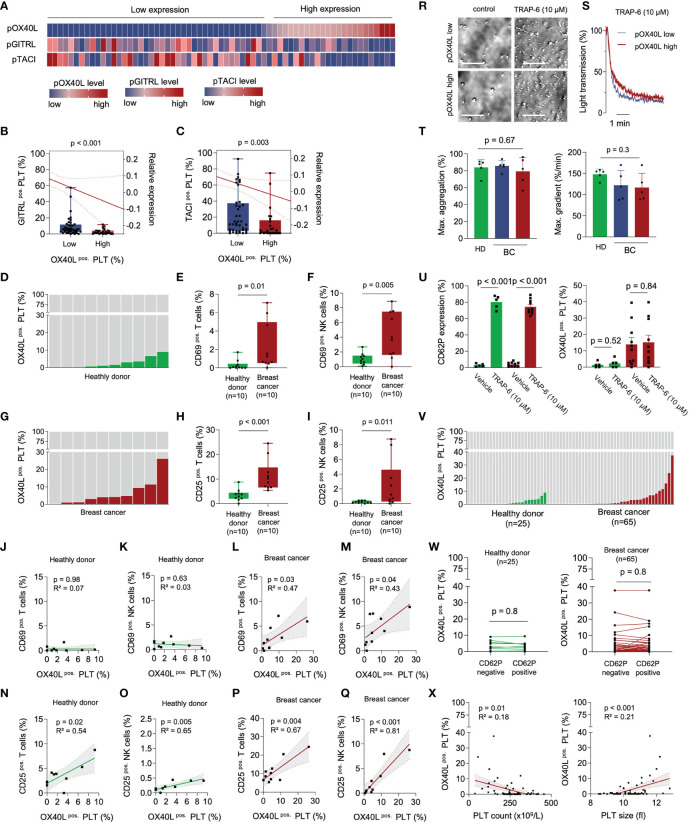
pOX40L in breast cancer patients is associated with a distinct platelet immunophenotype and activation of cytotoxic lymphocytes. **(A–C)** Comparative analysis of the platelet immune checkpoint molecules pOX40L, pTACI and pGITRL (n = 65). The gating strategy used to analyze pOX40L expression on platelets is given in **Supplementary Figure 3**. Co-expression of TACI **(B)** or GITRL (c) on pOX40L^low^ and pOX40L^high^ platelets, respectively. **(D–J)** Level of pOX40L in healthy donors **(D)** and breast cancer patients **(G)** and expression of CD69 **(E, F)** and CD25 **(H, I)** on NK cells and T cells of the respective donors (n = 10 each). **(J–Q)** Co-regulation of CD69/CD25 on NK/T cells and pOX40L in healthy donors and breast cancer patients (n = 10 each). r-t Comparative analysis of platelet activation in the presence or absence of TRAP-6 (10 µM) with regard to pOX40L status. Representative platelet aggregation studies **(R, S)** were performed in pOX40L high and low expressing platelets derived from breast cancer patients. Aggregation studies **(T)** were performed in platelets derived from 5 healthy donors (green) and 10 breast cancer patients (pOX40L low in blue, pOX40L high in red). **(U)** Expression of CD62P and OX40L on platelets from HD and breast cancer patients *ex vivo* (n = 10 each). **(V)** Expression of OX40L on platelets from healthy donors and the complete breast cancer cohort *ex vivo* (n = 25 and n = 65, respectively). **(W)** pOX40L level in healthy donors (HD), (left panel) and breast cancer patients (right panel) with regard to CD62P expression (n = 25 and n = 65, respectively). **(X)** Correlation of pTACI expression and platelet count (left panel) and platelet size (right panel). **(B, C)**, **(E, F)**, **(H, I)** Each dot represents a single patient. Boxes represent median and 25th to 75th percentiles, whiskers are minimum to maximum.

Although the expression of several immune checkpoint molecules on platelets has recently been described, their role in cancer is only poorly understood (23–25). In an effort to further investigate the impact of platelet-expressed immune checkpoints in solid tumors, we characterized the concurrent expression of the three TNF members pGITRL (pTNFSF18), pTACI (pTNFRSF13B), and pOX40L (pTNFSF4) in a cohort of breast cancer patients (**Figure 1A**). Interestingly we found a positive correlation of pGITRL and pTACI, two recently identified platelet-expressed immune checkpoint molecules with predictive value in breast cancer (24, 25). Despite the great homology between OX40 and GITR (26), the ligand of the immune stimulatory co-receptor OX40 on platelets was shown to be negatively correlated with pGITRL and pTACI expression (**Figures 1B,C**). This observation indicates that platelets express a plethora of different immune checkpoint molecules and the platelet immune phenotype (PIP) seems to be specifically regulated, especially in cancer. In in a previous study and in line with the different roles of GITR in NK cells and T cells, pGITRL was found to be associated with T cell but inversely correlated with NK cell activation in breast cancer (9, 24, 27). The fact that highest pGITRL level were present in intermediate tumor stages may support the notion that stimulation of T cell derived GITR contributes to tumor immunosurveillance of breast cancer but will lose its impact in furthering tumor progression in later stages. Expression of the receptor TACI on platelets is also specifically upregulated in breast cancer and is inversely correlated with metastasis and advanced tumor stage. It is not yet understood how pTACI may be involved in the (immune) regulation in breast cancer. One could speculate that pTACI induces reverse signaling into ligand-expressing cells such as breast cancer cells (28–30), but the existence of such signaling still warrants proof (31). Additionally, it was found that TACI is expressed on both, macrophages and tumor cells, particularly in breast cancer cases with poor prognosis (30). Thus, another explanation could be a transfer of TACI between platelets and malignant cells - which has been shown for other pathophysiologically relevant molecules like MHC class I and PD-L1 (19, 23) - or immune cells.

It is poorly understood how surface levels of immune regulatory molecules on platelets are regulated. Surface expression of some immunoregulative molecules like PD-L1 (23) and CD40L (32) for example are dependent on platelet activation. Changed expression levels in breast cancer however, might be regulated *via* trogocytosis, reprogrammed megakaryopoiesis (33) or protein synthesis in platelets which can occur at low levels (34). *In vitro* data show that soluble factors from tumor cells alter megekaryopoiesis in the MEG-01 cell line model and thus the megakaryocyte immunophenotype with regard to GITRL expression. One contributing factor appeared to be TGF-β which is known to be involved in the regulation of GITRL expression on dendritic cells (35). Alike, OX40L is expressed among others on antigen presenting cells where it is upregulated upon antigen presentation and costimulation including interferon gamma (IFN-γ) (36), and following stimulations with prostaglandin E2 (37), IL-18 (38) and thymic stromal lymphopoietin (TSLP) (39). Further studies are needed to study whether these factors also play a role in pOX40L regulation in the context of breast cancer.

Targeting of the co-stimulatory receptor OX40 is recently under evaluation as a novel strategy in cancer immunotherapy, and its expression on CD8+ cytotoxic T cells of breast cancer patients was found to be upregulated upon neo-adjuvant chemotherapy (40). When comparatively analyzing pOX40L and the immunophenotype in a screening cohort of healthy donors and breast cancer patients, we found higher pOX40L levels to be associated with increased T and NK cell activation levels (**Figures 1D–I**). Especially in breast cancer patients, CD69 and CD25 expression on T and NK cells were strongly correlated with the expression of OX40L on platelets (**Figures 1J–Q**). This is particularly interesting since previous studies report on an enlarged CD4+ FOXP3+ T cells population in breast cancer (41, 42). Yet, further work is required to evaluate whether pOX40L-dependent signaling contributes to the expansion of regulatory T cells. Taking the known immune-activating role of OX40L on other cell types, including B cells, mast cells or endothelial cells into account, our observation might point to pOX40L as a novel and underestimated player mediating immune cell activation and anti-tumor immunity.

### Regulation of OX40L on Platelets During Platelet Activation

As it is well established that the platelet surface proteome, as well as the PIP, is modulated *via* platelet activation, we further analyzed pOX40L in the context of platelet reactivity. Platelet aggregometry showed comparable functional capabilities in the presence of the PAR-1 agonist TRAP-6 independent of pOX40L expression (**Figures 1R–T**). This confirms previous data showing that the expression of immune checkpoint molecules on platelets is not associated with platelet reactivity (23, 24). In contrast to previous observations, platelet activation *ex vivo* did not result in an expression change of pOX40L on the platelet surface (**Figure 1U**). Confirming this observation *ex vivo*, we found no significant correlation of pOX40L and CD62P in our entire cohort of healthy donors and breast cancer patients (**Figures 1V, W**). Since pOX40L surface expression seems to be rarely regulated during platelet activation, complex adjustment algorithms to model surface expression *in vivo* are subsequently not required. This highlights pOX40L as a novel putative biomarker candidate in breast cancer. We found that pOX40L, similar to pTACI and pGITRL, was negatively correlated with platelet count and associated with larger platelet size (**Figure 1X**). This might suggest a complex interplay of anti-cancer treatment, platelet regeneration, and tumor homeostasis, resulting in a subsequent chronic inflammatory environment might be involved in PIP regulation, including pOX40L (43, 44).

### Regulation of pOX40L Expression During Breast Cancer Progression

**Figure 2:**
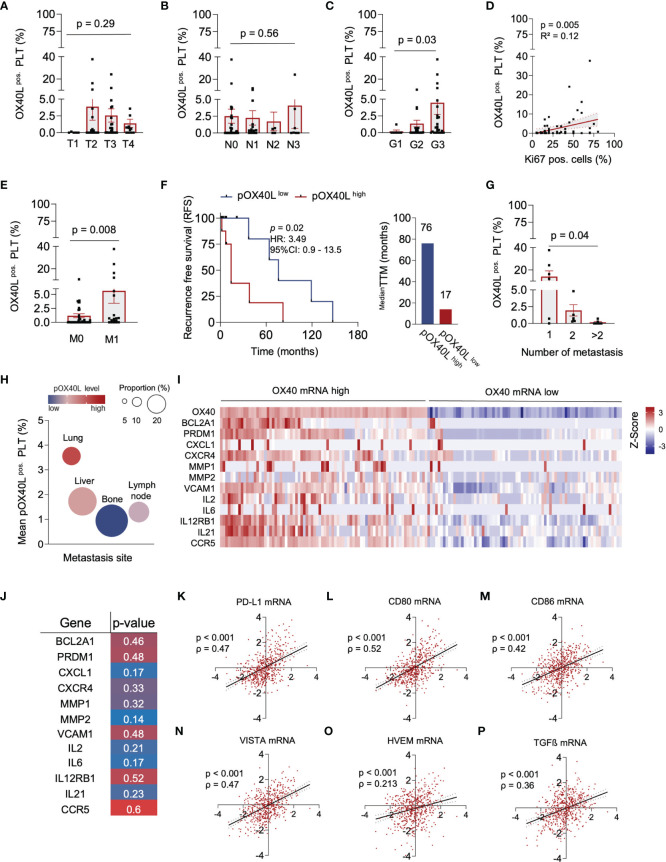
| pOX40L predicts disease relapse and metastasis in breast cancer. **(A)** pOX40L expression in different tumor stages (T), (n = 65) **(B)** Association of pOX40L expression and lymph node invasion (N), (n = 65). **(C)** pOX40L level in patients with different tumor grading **(G)**, (n = 65). **(D)** Correlation between pOX40L expression on platelets of 65 BC patients and the Ki67 positive cells (%). **(E)** pOX40L expression in patients with and without metastatic disease (M), (n = 65). **(F)** Kaplan–Meier curves estimates of RFS (months) in patients with a pOX40L > mean (pOX40L^high^ = red) and pOX40L level < mean (pOX40L^low^ = blue), (n = 65). Median time to metastasis (pOX40L^high^ = red, pOX40L^low^ = blue). **(G)** pOX40L expression in patients with different number of metastasis (n = 65). **(H)** Mean expression of pOX40L in different metastatic organs (n = 20). **(I)** Heatmap depicting the relative mRNA level OX40 and a gene set associated with lung metastasis, anti-apoptotic and tumor-promoting signatures in breast tumors (TCGA-Firehose legacy), (n = 65). **(J)** Spearman correlation mRNA gene signatures and OX40 mRNA. **(K–P)** Scatter plot of OX40 mRNA and PD-L1 (**K)**, CD80 (l), CD86 **(M)**, VISTA **(N)**, HVEM **(O)** and TGFß **(P)** mRNA level in breast cancer. Each dot represents a single patient. **(A–E, G, K–P)** Each dot represents a single patient. **(A–E, G)** Data are mean ± SEM.

**Figure 3:**
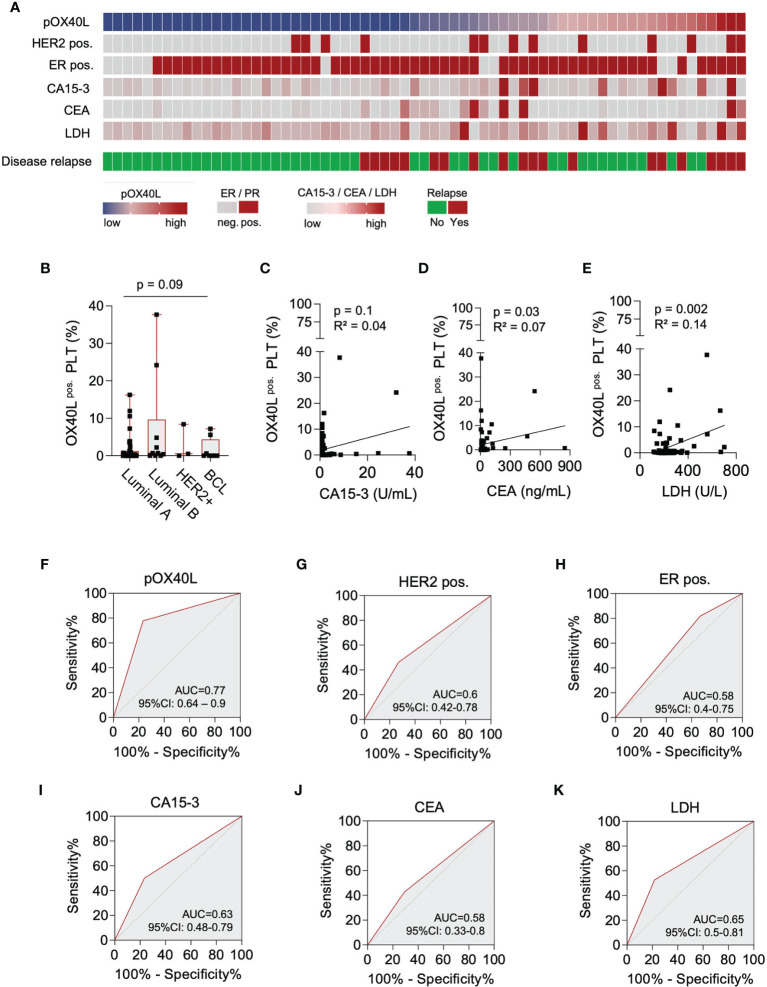
Predictive value of pOX40L in breast cancer relapse. **(A)** Comparative analysis of pOX40L and the clinical markers HER2, ER, CA 15-3, CEA and LDH. **(B)** Association of pOX40L expression and different breast cancer subtypes. **(C-E)** Correlation of pOX40L and conventional tumor marker including CA15-3 **(C)**, CEA **(D)** and LDH **(E)**. **(F–K)** Predictive value of pOX40L **(F)**, HER2 **(G)**, ER **(H)**, CA15-3 **(I)**, CEA **(J)** and LDH **(K)** expression for disease relapse was analyzed using ROC (Area under the ROC curve) analysis.

In the next step, we studied the association of pOX40L with clinical parameters and disease progression. While pOX40L status was irrespective of the tumor stage (T) and lymph node invasion (N), it was associated with histological grading (G) (*p* = 0.03), whereas most pOX40L^high^ patients were found in the G3 group (**Figures 2A–C**). In line, the proliferation index (Ki67) of tumors and formation of metastasis, which both constitute negative prognostic markers for patients, were directly associated with higher pOX40L levels in breast cancer patients (p = 0.005 and p = 0.008), respectively (**Figures 2D, E**). Accordingly, with a median time to metastasis (TTM) of 17 months compared to 76 months, recurrence-free survival (RFS) was significantly shorter in patients with a high pOX40L expression compared to patients showing low levels of pOX40L (p=0.02) (**Figure 3F**). Interestingly, this observation seems to be in contrast to the proposed role of pOX40L as an immune-stimulating player promoting anti-tumor effects. Of note, we did not observe any correlation of pOX40L expression and different systemic treatments or number of received treatment regimens (**Supplementary Figure1 A-C**).

We observed that pOX40L status was inversely correlated with the number of metastatic organs. While patients with high levels of pOX40L showed only one metastatic site, low pOX40L levels were associated with a higher metastatic burden in multiple organs (**Figure 2G**). These findings led to the hypothesis that pOX40L might be involved in tumor immune cell interactions. Interestingly, pOX40L expression was shown to be independent of treatment modality, kind of treatment and number of treatment regimen (**Supplementary Figure 1 A-C**). When analyzing the distribution of metastasis in our cohort we observed that patients with high expression of pOX40L frequently developed lung metastasis (**Figure 2H**). Following this observation, we further investigated putative OX40/OX40L signaling in malignant cells. We correlated *OX40* mRNA expression in a TCGA breast cancer data set (TCGA, Firehose Legacy) and gene-expression signatures in breast cancer associated with lung metastasis (45). Remarkably, the main genes involved, e. g. MMP1, MMP2, SPARC, VCMAM1, CXCL1, and CXCR4 were found to be significantly associated in with high *OX40* mRNA expression (**Figure 2I**). In addition, these genes clustered with the expression of anti-apoptotic and tumor-promoting factors (**Figures 2I,J**). Finally, we studied whether additional immune checkpoint molecules might be co-regulated with *OX40* mRNA in breast cancer cells. We found a strong correlation of OX40 and PD-L1, CD80, CD86, VISTA, HVEM, and TGFß, hypothesizing that OX40 expression on tumor cells might be associated with an immune-inhibitory phenotype (**Figures 2K–P**).

Our data suggest that OX40L and OX40 are expressed on various cell types in the tumor microenvironment, and OX40/OX40L signaling in cancer might be much more complex than previously known. This might be reflected by the limited success of OX40 targeting therapies so far (10–12). As a result, further comprehensive studies investigating both, OX40 signaling in tumor cells and the functional role of pOX40L on platelets are needed. Since expression of OX40 on breast cancer cells has already been shown to be associated with an aggressive cancer phenotype (46), expression of OX40 on tumor tissue as well as determination of pOX40L might be useful to stratify patients who might benefit from an immunotherapy targeting the OX40 pathway.

Since pOX40L is not regulated upon platelet activation and was shown to predict PFS in our cohort, we finally investigated the potential of pOX40L as a prognostic marker in breast cancer. We comparatively analyzed pOX40L expression and commonly used clinical markers (47–49) to predict disease relapse in breast cancer (**Figure 3A**). Whereas pOX40L expression, as well as CA15-3, CEA and LDH was independent of the respective subtype (**Figure 3B**; **Supplementary Figures 2A–C**), we observed a positive correlation of pOX40L expression and the conventional tumor markers CEA and LDH (**Figures 3C–E**). This in line with our observation that pOX40L was also associated with higher Ki67 level and higher tumor grading. Of note, with a sensitivity of 0.78 (95% CI: 0.55 – 0.91) and specificity of 0.77 (95% CI: 0.63 – 0.86) pOX40L showed the highest accuracy in predicting disease relapse (**Figures 3F–K**). Since ER/HER2 status represents an indispensable part of the management of breast cancer patients we additionally analyzed the influence of ER and HER2 status on pOX40L expression in our cohort. Interestingly we didn’t observe any correlation of hormone receptor or HER2 status and expression of OX40L on platelets (**Supplementary Figures 2D, E**). However, taking into account that triple negative and HER2-positive breast cancer patients present the highest levels of tumor infiltrating lymphocytes (TILs) (50), which is prerequisite for the successful use of immunotherapy, pOX40L expression as well as HER2 and hormone receptor status might be useful predictive tools for OX40 agonists. Of note, additional data are needed to further investigate such an approach.

Nevertheless, our exploratory study suffers from some limitations. Due to the fact that we used an exploratory study design our breast cancer cohort constitutes an unequal representation of tumor stages, different treatments and different cycles of various treatment regimens. Even if pOX40L expression seems to be independent of tumor stages, lymph node invasion, treatment modalities and number of treatment regimen (**Supplementary Figures 1A–C**), further consecutive analysis in a balanced, prospective study cohort are needed.

In addition, our exploratory study was not powered to compare the determination of pOX40L with conventional screening methods including mammogram/MRI or determination of CEA or CA15-3. Further prospective data are needed to verify the predictive role of pOX40L in breast cancer. Nevertheless, since high level of pOX40L were associated with a shorter RFS and TTM, determination of pOX40L might be a useful, noninvasive and cost-effective method allowing for longitudinal follow-up analysis. In patients with high levels of pOX40L follow-up care might be intensified and supplemented by specific screening for metastases.

In conclusion, our explorative data not only highlight the prognostic potential of the platelet-expressed immunoregulatory molecule OX40L as a biomarker in breast cancer progression. They further indicate that OX40/OX40L signaling in cancer seems to be complex and might play a controversial role in tumor biology *via* orchestrating immune activation and tumorigenesis, an approach which requires further functional and clinical validation.

## Material and Methods

### Patients

During 2019-2021, 65 breast cancer patients treated at the Department of Obstetrics and Gynecology and the Department of Medical Oncology and Pneumology were included in our prospective study. The study was approved by IRB (ethics committee of the Faculty of Medicine of the Eberhard Karls Universitaet Tuebingen) and of the University Hospital (13/2007V). Written informed consent in accordance with the Helsinki protocol was given in all cases. The patient characteristics in detail are given in **Supplementary Table 1**.

### Reagents

Paraformaldehyde was from Affymetrix (Santa Clara, CA, USA). Anti-OX40L antibodies were from Ancell Corporation (Bayport, MN, USA). Anti-human TACI, anti-human GITRL antibody and the respective isotype control were from R&D Systems (Minneapolis, MN). CD41a-PeCy5, and CD62P-FITC were from BD Pharmingen, CD3-APC/Fire, CD69-PE and CD25-FITC and CD56-PECy7 were obtained from BioLegend. The goat anti-mouse PE conjugate was from Dako (Glostrup, Denmark). Dead cells were excluded using Fixable Aqua (Invitrogen, Carlsbad, CA, USA) after extracellular staining according to the manufacturer’s instructions. Bicoll Separating Solution was purchased from Biochrom AG (Berlin, Germany).

### Platelet Aggregation

Platelet aggregation was analyzed using the four-channel light transmission platelet aggregometer APACT 4004 (Elitech, Puteaux, France) according to the manufacturer’s instructions. For platelet activation *ex vivo* 10 µM of Thrombin Receptor Activator Peptide 6 (TRAP-6) was added to the platelets.

### Generation and Preparation of Peripheral Blood Mononuclear Cells (PBMC) and Peripheral Blood Platelets

PBMCs were obtained from patients and healthy donors after informed writing consent. Preparation of PBMCs was performed as previously described (19). Peripheral blood platelets were collected in citrate buffer and briefly centrifuged for 20 min at 120×g. For the generation of platelet-rich plasma, platelets were washed with citrate wash buffer (128 mmol/L NaCl, 11 mmol/L glucose, 7.5 mmol/L Na2HPO4, 4.8 mmol/L sodium citrate, 4.3 mmol/L NaH2PO4, 2.4 mmol/L citric acid, and 0.35% bovine serum albumin).

### Flow Cytometry

Flow cytometry was performed as described previously (24). The percentage of positive cells was calculated as follows: “% surface expression obtained with specific antibody” − “% surface expression obtained with isotype control”. T cells were selected by positivity for CD3, and NK cells by a CD56+CD3− phenotype. Platelets were selected by CD41a+ and CD62P− (resting) or CD62P+ (activated).

### Statistics

For continuous variables student’s t test, Mann-Whitney U test, one-way ANOVA and Kruskal-Wallis test was used. For multiple comparison Dunn’s multiple comparison test was used. For categorical data we used chi‐squared test or Fisher’s exact test. Correlation of pOX40L expression and CD69/CD25 expression on T and NK cells, platelet size and platelet volume as well as Ki67 level and pOX40L expression was analyzed using simple linear regression analysis. Correlation of mRNA expression was performed using simple linear regression analysis. Recurrence free survival (RFS) were calculated using the Kaplan‐Meier method. Hazard ratios (HRs) were determined using Cox regression analysis. High pOX40L expression was defined as follows: pOX40L high > mean p=OX40L, pOX40L low < mean pOX40L. The predictive value of pOX40L was evaluated by examining the area under the receiver‐operator characteristic (ROC) with a confidence interval of 95%. All statistical tests were considered statistically significant when P was below 0.05. Statistical analysis was performed using GraphPadPrism (v.8.4.0).

## Data Availability Statement

The genome dataset presented in this study can be found in online repositories (The Cancer Genome Atlas Database (TCGA), https://www.cbioportal.org). The data that support the findings of this study are available from the corresponding author, SM upon reasonable request.

## Ethics Statement

The study was approved by IRB (ethics committee of the Faculty of Medicine of the Eberhard Karls Universitaet Tuebingen) and of the University Hospital (13/2007V). The patients/participants provided their written informed consent to participate in this study.

## Author Contributions

CH, SM, LZ, and HS conceived and designed the study. ML, KC, and YZ conducted *in vitro* experiments. AK and AH conducted patient data and sample collection as well as medical evaluation and analysis. SR, MH, KK, SM, and CH analyzed data and performed statistical analyses. SR, CH, and SM prepared figures and tables; SR, SM, and CH wrote the first draft of the manuscript. HS, LZ, and AH contributed to data interpretation and manuscript edit. All authors critically reviewed, read, and approved the final manuscript.

## Funding

This work was funded by the Deutsche Forschungsgemeinschaft (DFG, German Research Foundation) under Germany’s Excellence Strategy - EXC 2180 - 39090067 and DFG, project number 374031971–TRR 240/project B05. SM was supported by the Institutional Strategy of the University of Tuebingen (Deutsche Forschungsgemeinschaft, ZUK 63) and the Deutsche Forschungsgemeinschaft, MA 8774/1-1. HS was funded by Deutsche Forschungsgemeinschaft, SA1360/7-3, Wilhelm Sander-Stiftung, 2007.115.3, and Deutsche Krebshilfe 70112914. SR is participant in the BIH Charité Clinician Scientist Program funded by the Charité – Universitaetsmedizin Berlin, and the Berlin Institute of Health at Charité (BIH).

## Conflict of Interest

The authors declare that the research was conducted in the absence of any commercial or financial relationships that could be construed as a potential conflict of interest.

## Publisher’s Note

All claims expressed in this article are solely those of the authors and do not necessarily represent those of their affiliated organizations, or those of the publisher, the editors and the reviewers. Any product that may be evaluated in this article, or claim that may be made by its manufacturer, is not guaranteed or endorsed by the publisher.

## Supplementary Material

Supplementary Figure 1 Association of pOX40L and breast cancer treatment. **(A)** Comparative analysis of pOX40L and different breast cancer subtypes. **(B)** Association of pOX40L and endocrine therapy. **(C)** Correlation of pOX40L and different numbers of treatment regimen.

Supplementary Figure 2 pOX40L expression in different breast cancer subtypes Expression of CA15-3 **(A)**, CEA **(B)** and LDH **(C)** in different breast cancer subtypes. **(D)** pOX40L expression in HER2 positive and negative patients. **(E)** pOX40L expression in ER positive vs. ER negative patients. pos. = positive, neg. = negative.

Supplementary Figure 3 Gating strategy used to analyze pOX40L expression on platelets pOX40L levels were assessed by flow cytometry. Platelets were defined as CD41a-positive subcellular fragments and specific staining for OX40L level was analyzed in association with the platelet activation marker P-selectin (CD62P). If not indicated otherwise, data reflect pOX40L expression on all CD41+ platelets, i.e. activated (CD62P+) and resting (CD62P-) platelets.

Supplementary Table 1 Patient characteristics. Other immune checkpoint molecules in parts of this cohort have been published previously (24, 25, 51).
