# Supplementary material for: Controversial Role of the Immune Checkpoint OX40L Expression on Platelets in Breast Cancer Progression
Source: Front Oncol. 2022 Jul 8;12:917834. doi: 10.3389/fonc.2022.917834 (PMC9304936; doi:10.3389/fonc.2022.917834)
Supplement: Supplementary Figure 1 — Association of pOX40L and breast cancer treatment. (A) Comparative analysis of pOX40L and different breast cancer subtypes. (B) Association of pOX40L and endocrine therapy. (C) Correlation of pOX40L and different numbers of treatment regimen. [file Image_1.pdf]

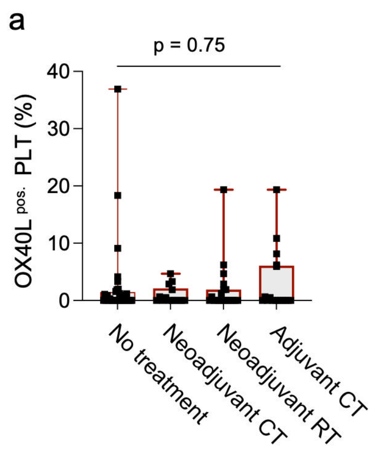

Suppl. Figure 1: Association of pOX40L and breast cancer treatment
a Comparative analysis of pOX40L and different breast cancer subtypes. b Association of pOX40L and endocrine therapy. c Correlation of pOX40L and different numbers of treatment regimen.
