# Supplementary material for: Controversial Role of the Immune Checkpoint OX40L Expression on Platelets in Breast Cancer Progression
Source: Front Oncol. 2022 Jul 8;12:917834. doi: 10.3389/fonc.2022.917834 (PMC9304936; doi:10.3389/fonc.2022.917834)
Supplement: Supplementary Figure 2 — pOX40L expression in different breast cancer subtypes Expression of CA15-3 (A), CEA (B) and LDH (C) in different breast cancer subtypes. (D) pOX40L expression in HER2 positive and negative patients. (E) pOX40L expression in ER positive vs. ER negative patients. pos. = positive, neg. = negative. [file Image_2.pdf]

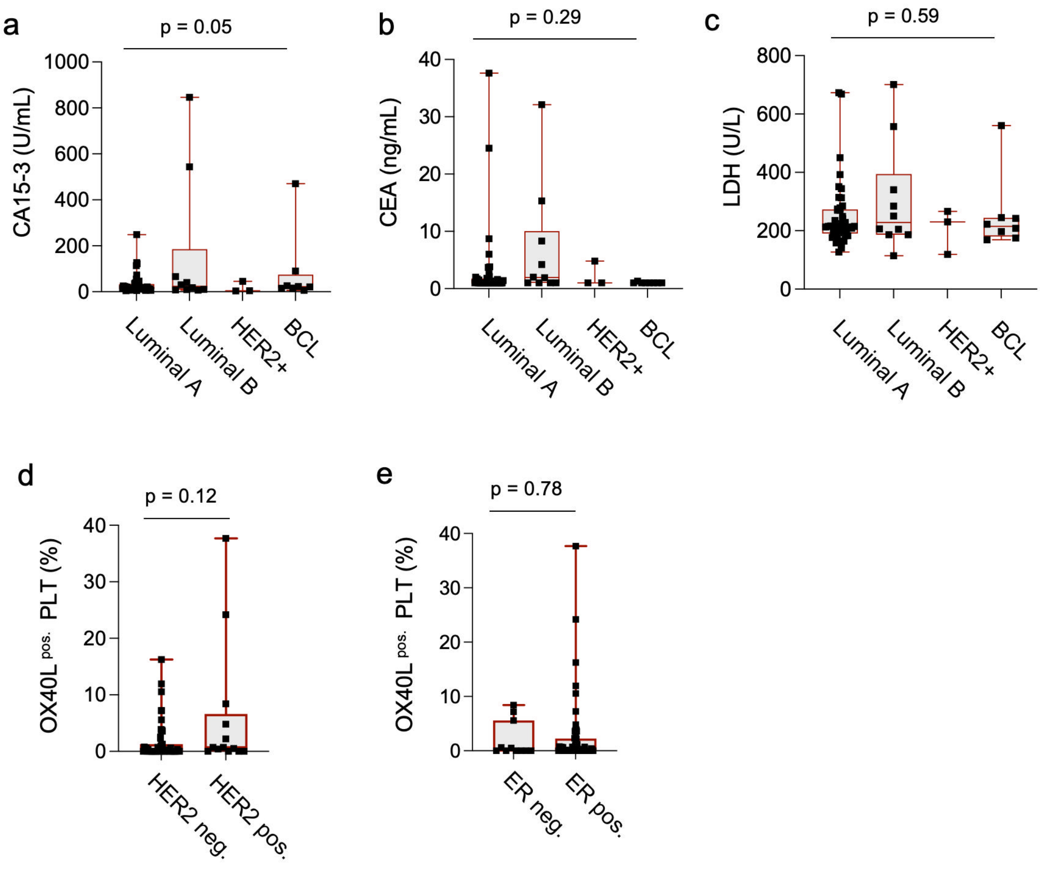

Suppl. Figure 2: pOX40L expression in different breast cancer subtypes
Expression of CA15-3 (a), CEA (b) and LDH (c) in different breast cancer subtypes. d pOX40L expression in HER2 positive and negative patients. e pOX40L expression in ER positive vs. ER negative patients. pos. = positive, neg. = negative.
