# Supplementary material for: Controversial Role of the Immune Checkpoint OX40L Expression on Platelets in Breast Cancer Progression
Source: Front Oncol. 2022 Jul 8;12:917834. doi: 10.3389/fonc.2022.917834 (PMC9304936; doi:10.3389/fonc.2022.917834)
Supplement: Supplementary Figure 3 — Gating strategy used to analyze pOX40L expression on platelets pOX40L levels were assessed by flow cytometry. Platelets were defined as CD41a-positive subcellular fragments and specific staining for OX40L level was analyzed in association with the platelet activation marker P-selectin (CD62P). If not indicated otherwise, data reflect pOX40L expression on all CD41+ platelets, i.e. activated (CD62P+) and resting (CD62P-) platelets. [file Image_3.pdf]

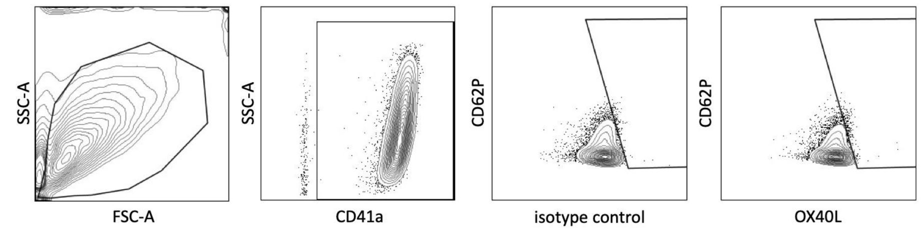

Suppl. Fig. 3: Gating strategy used to analyze pOX40L expression on platelets
pOX40L levels were assessed by flow cytometry. Platelets were defined as CD41a-positive subcellular fragments and specific staining for OX40L level was analyzed in association with the platelet activation marker P-selectin (CD62P). If not indicated otherwise, data reflect pOX40L expression on all CD41+ platelets, i.e. activated (CD62P+) and resting (CD62P-) platelets.
