# Supplementary material for: Controversial Role of the Immune Checkpoint OX40L Expression on Platelets in Breast Cancer Progression
Source: Front Oncol. 2022 Jul 8;12:917834. doi: 10.3389/fonc.2022.917834 (PMC9304936; doi:10.3389/fonc.2022.917834)
Supplement: Supplementary Table 1 — Patient characteristics. Other immune checkpoint molecules in parts of this cohort have been published previously (24, 25, 51). [file Table_1.docx]

**Supplementary Table 1**

**Patient characteristics**

| **Patient characteristics** | **Total** |
| --- | --- |
|  | (n=65) |
| **Gender** |  |
| female sex, n (%) | 65 (100) |
| **Age** |  |
| Age in years, mean–yr.±SD  (range) | 60.6 ± 13.1  (27 to 87) |
|  |  |
| **TNM classification, n (%)** |  |
| Stage |  |
| T1 | 5 (7.7) |
| T2 | 20 (30.8) |
| T3 | 28 (43.1) |
| T4 | 12 (18.5) |
| Node |  |
| N0 | 39 (60) |
| N1 | 14 (21.5) |
| N2 | 5 (7.7) |
| N3 | 7 (10.8) |
| Metastasis |  |
| M0 | 45 (75.7) |
| M1 | 20 (30.8) |
|  |  |
| **Localization of primary tumor** |  |
| Right | 23 (69.2) |
| Left | 42 (64.6) |
|  |  |
| **Histological grading, n (%)** |  |
| G1 | 6 (9.2) |
| G2 | 33 (50.8) |
| G3 | 26 (40) |
|  |  |
| **ER positive, n (%)** | 54 (83.1) |
| **Her2 positive, n (%)** | 13 (20) |
|  |  |
|  |  |
|  |  |
